# Confocal spectral microscopy, a non-destructive approach to follow contamination and biofilm formation of mCherry *Staphylococcus aureus* on solid surfaces

**DOI:** 10.1038/s41598-021-94939-2

**Published:** 2021-08-02

**Authors:** Muhammad Tanveer Munir, Nattar Maneewan, Julien Pichon, Mohammed Gharbia, Ismael Oumarou-Mahamane, Jessica Baude, Chantal Thorin, Didier Lepelletier, Patrice Le Pape, Matthieu Eveillard, Mark Irle, Hélène Pailhoriès, Florence Aviat, Christophe Belloncle, Michel Federighi, Laurence Dubreil

**Affiliations:** 1 LIMBHA, Ecole Supérieure du Bois, 44000 Nantes, France; 2 UMR703 PAnTher APEX, INRAE/ONIRIS - La Chantrerie, 101 Route de Gachet, 44307 Nantes, France; 3 UMR INRAE 1014 SECALIM, Oniris, 44307 Nantes, Cedex 03, France; 4 CIRI, Inserm U1111, Lyon 1 University, ENS Lyon, CNRS UMR 5308, Lyon, France; 5 NP3 Laboratory, Oniris, Nantes, France; 6 MiHAR EE 1701 S, IRS 2, University of Nantes, 44200 Nantes, France; 7 EA 1155 IICiMed, IRS 2, University of Nantes, 44200 Nantes, France; 8 CRCINA, Inserm, University of Nantes, University of Angers, 44200 Angers, France; 9 Laboratory of Bacteriology-Hygiene, University Hospital of Angers, 49933 Angers, France; 10 HIFIH, UPRES EA3859, SFR 4208, University of Angers, Angers, France; 11 Your ResearcH-Bio-Scientific, 44430 Le Landreau, France

**Keywords:** Confocal microscopy, Risk factors, Bacterial infection, Applied microbiology

## Abstract

Methods to test the safety of wood material for hygienically sensitive places are indirect, destructive and limited to incomplete microbial recovery via swabbing, brushing and elution-based techniques. Therefore, we chose mCherry *Staphylococcus aureus* as a model bacterium for solid and porous surface contamination. Confocal spectral laser microscope (CSLM) was employed to characterize and use the autofluorescence of Sessile oak (*Quercus petraea*), Douglas fir (*Pseudotsuga menziesii*) and poplar (*Populus euramericana alba* L.) wood discs cut into transversal (RT) and tangential (LT) planes. The red fluorescent area occupied by bacteria was differentiated from that of wood, which represented the bacterial quantification, survival and bio-distribution on surfaces from one hour to one week after inoculation. More bacteria were present near the surface on LT face wood as compared to RT and they persisted throughout the study period. Furthermore, this innovative methodology identified that *S. aureus* formed a dense biofilm on melamine but not on oak wood in similar inoculation and growth conditions. Conclusively, the endogenous fluorescence of materials and the model bacterium permitted direct quantification of surface contamination by using CSLM and it is a promising tool for hygienic safety evaluation.

## Introduction

Wood is an organic and porous material that is abundantly used in hygienically important places, such as in the architecture or indoor construction of the interiors of healthcare and residential buildings, and in the food industry as contact surfaces for food preparation, packaging and storage. This material is the most preferred for its naturalness and eco-friendly nature and may have restorative effects on the psycho-physiological health of building occupants^1^.

Microbial presence on solid surfaces inside the healthcare institutes poses a continuous cross-contamination risk, especially, when the microbes can remain hidden in porous surfaces after cleaning^2^. Therefore, the use of porous materials, such as wood in hygienically important places such as healthcare institutions and food industry, is often questioned owing to the lack of sufficient evidence on its hygienic properties and safety. Especially regarding the hygienically important microbes such as *Staphylococcus aureus*, which is one of the most common bacteria responsible for hospital-acquired infections (HAI)^3^ and foodborne outbreaks^4^. This bacterium survives on inanimate surfaces from days to months depending upon environmental conditions^5–8^.

Studies have shown that wood materials may have antimicrobial properties against a wide range of hygienically important microbes^9^, including *S. aureus*^3,10^. This microbe countering behaviour of wood is generally attributed to its chemical composition of extractives, such as flavonoids and phenolics, which have an inhibitory role against microbial growth^11^. In addition, the survival of different bacteria and fungi has also been reported to be lower on wood as compared to other inanimate materials^11,12^. Therefore, the physical structure of wood is also regarded as a component of antimicrobial activity of wood^9,11^. However, it is not clear if this physical effect is merely an underestimation of microbial recovery done using swabbing, brushing, gringing and elution-based methods^13^. Therefore, studying the microbial distribution inside the porous wood material will provide clues whether the microbes can attach and stay hidden inside the surfaces or are killed due to the antimicrobial activity of wood.

The microscopic tools could be very promising to study morphology, spatial distribution, survival and viability of microbes on different surfaces^14–16^. However, there are certain limitations on their application to porous surfaces without damaging the surface integrity of substrate^17^. Scanning Electron Microscopy (SEM) is widely used to observe the presence of microbial contaminants on wood surfaces^13,18^. SEM is restricted, however, to 2D exploration, and thus the 3D observation of microbial colonization inside the pores and cracks of wood is very difficult^19^. It requires a series of highly invasive fixation steps that is incompatible with live imaging and is thus unable to provide direct information on the microbial survival on analysed porous substrates like wood^16^. Avoiding the fixation steps in the direct microscopic approaches such as in environmental SEM can modify the morphology of wood samples and the microbes^17,20^. Therefore, other more direct microscopic tools are sought to visualize the microbial viability and spatial distribution inside the wooden structures. Confocal scanning laser microscopy (CSLM) has been described as a potent non‐invasive optical sectioning tool^19^. This tool can observe the micro-morphologies of microbe interaction within the porous material at a depth of around 50 µm without incising samples^17^.

The present work aims to develop new tools to investigate bio-distribution of *S. aureus* on untreated wooden surfaces with different incubation times and to study biofilm formation on different materials used for healthcare buildings or food contact surfaces.

## Results

This study aimed to develop a methodology to visualize firstly the spatial distribution of the *S. aureus* bacterium on different wood species to be able to analyse the bacterial contamination on wooden surfaces over time and secondly to apply this methodology to investigate biofilm formation potential of bacterium on wood as compared to melamine.

### Autofluorescence of wood

**Figure 1:**
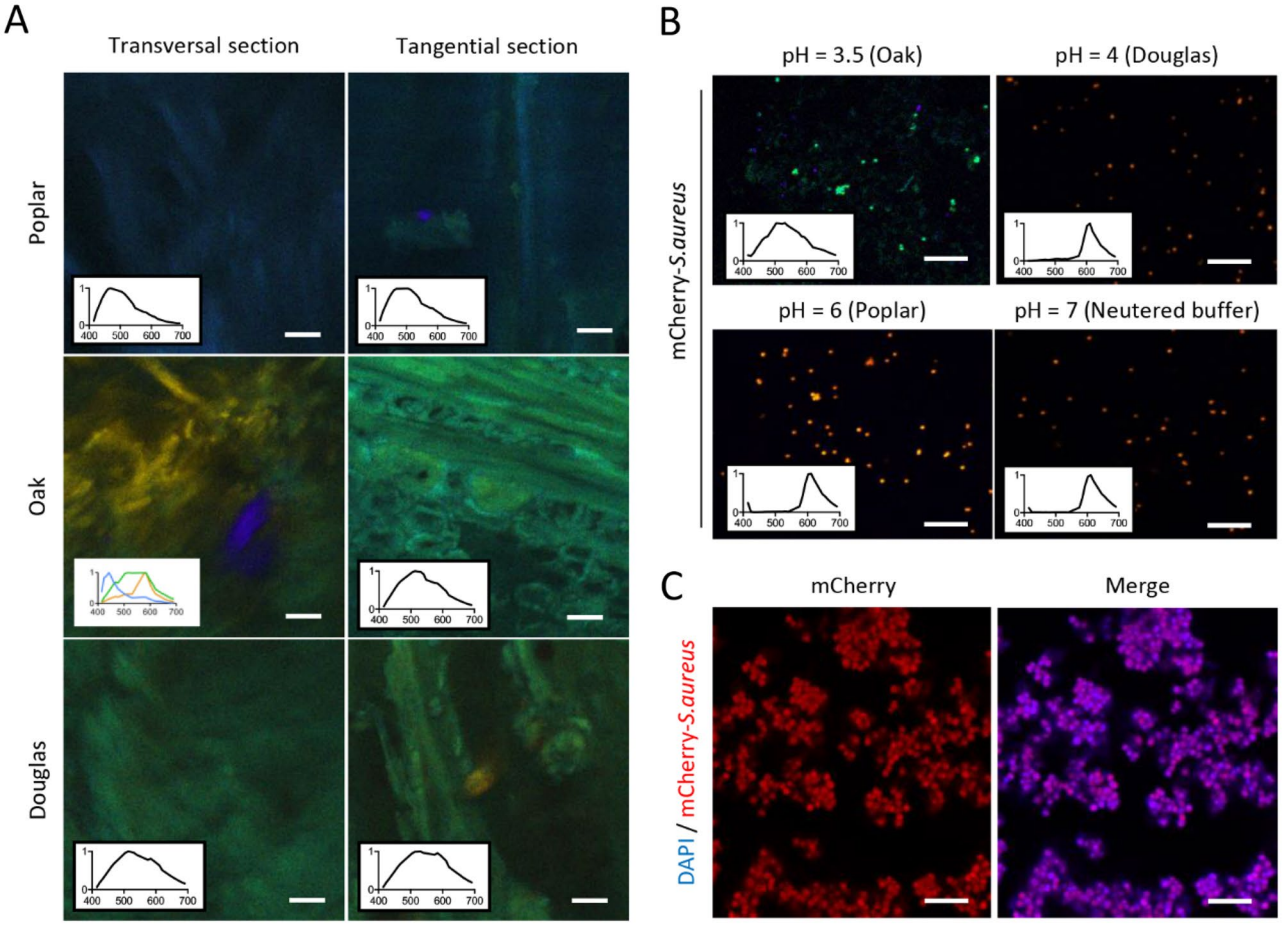
Wood imaging using spectral microscopy. **(A)** Spectral imaging of poplar, oak and Douglas fir autofluorescence (respectively, top, middle and bottom) with transversal (left) and tangential (right) section. Acquisition was performed with spectral confocal microscope (excitation at 405 nm, fluorescence spectra collection from 410 to 690 nm). The colors in the images in the Fig. 1A are pseudocolors which represented wavelengths emission. The mean spectra obtained from the images showed a large emission spectra between 400 nm (blue) and 700 nm (red) with more or less fluorescence in blue, green and yellow dependant of wood species and section orientation. 3 different spectra has ben assigned to oak RT section. Scale bar = 20 µm. (B) Spectral imaging of mCherry—*Staphylococcus aureus* at pH = 3.5 (oak pH), 4 (Douglas fir pH), 6 (poplar pH) and 7 (neutral pH). Scale bar = 5 µm. (C) Confocal microscopy of mCherry—*S. aureus* (red) solution prepared in PBS for wood inoculation. All bacteria are stained with DAPI (blue). Scale bar = 5 µm.

Measurements were done on wood disc by using 405 nm excitation and fluorescence collection between 405 and 690 nm. The autofluorescence spectra of wood material was quite large with emission wavelengths ranging from 435 to 690 nm (Fig. 1). These measurements were done both for RT and LT sections and for the three species of wood (Sessile oak, Douglas fir and poplar). The shape of spectra obtained from different wood species was relatively common except for oak RT section with a peak at 590 nm. The spectral difference between oak RT and LT sections highlighted that different fluorescent components could be visible due to the orientation of the section. These spectra were used as reference spectra in further measurements to separate wood fluorescence from bacterial fluorescence.

Effect of acidic pH on fluorescence properties of fluorescent protein mCherry of *S. aureus* was studied at pH of 3.5, 4 and 6. These values were chosen because the ground wood samples of oak, Douglas fir and poplar had pH values of 3.8, 4.4, 6.4, respectively^21^.

The fluorescence mCherry spectra observed at different pH are given in Fig. 1B. According to the literature, the fluorescence maximum emission peak of mCherry was obtained at 610 nm between pH 4 and pH 10^22^, whereas a dramatic shift of mCherry spectra was observed at pH 3.5 with a peak at 500 nm.

Therefore, to avoid the modification of mCherry spectra fluorescence in the presence of acidic medium, mCherry *S. aureus* inoculation experiments were performed in the presence of phosphate buffer saline (PBS), (i) to prepare a bacterial suspension, (ii) to store wood disc after fixation until observation with confocal microscopy. The suspension of mCherry *S. aureus* was stained with DAPI to assess the level of *S. aureus* transfection from plasmidic construction. Hundred percent of the bacterial cells were both blue and red-stained, it was demonstrated by “overlap analysis” on threshold images by using ImageJ (https://imagej.nih.gov/ij/).

### 3D microbial distribution on wood

The LT and RT faces of wood have very different porosities. In general, RT surface is more porous compared to LT sections. Therefore, the distribution of microbes is also expected to vary depending upon the porosity of surface. For this study, we did not measure the surface roughness of these samples, but in general the RT cuts were rougher than LT. The height between the highest surface point of the wood vs. the lowest valley was 10 μm and after that we observed at least 20 μm in depth. Penetration of the laser was limited by the solid wood density.

**Figure 2:**
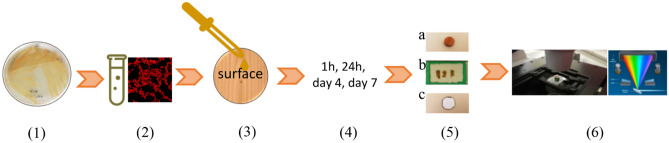
Experimental protocol used to characterize *S. aureus* bio-distribution on wood surface using spectral confocal laser microscopy (CSLM). Experimental steps: (**1**) Freshly cultured mCherry—*S. aureus,* (**2**) bacterial suspension prepared at 1.7 McFarland in PBS, (**3**) inoculation of wood disc surface, (**4**) different incubation time in dark, at room temperature, (**5) (a)**—wood disc on glass slide for direct non-destructive observation, (**b**)—wood section mounting on slide in PBS, (**c**)—melamine piece on glass slide for direct observation. (**6**) CSLM imaging.

**Figure 3:**
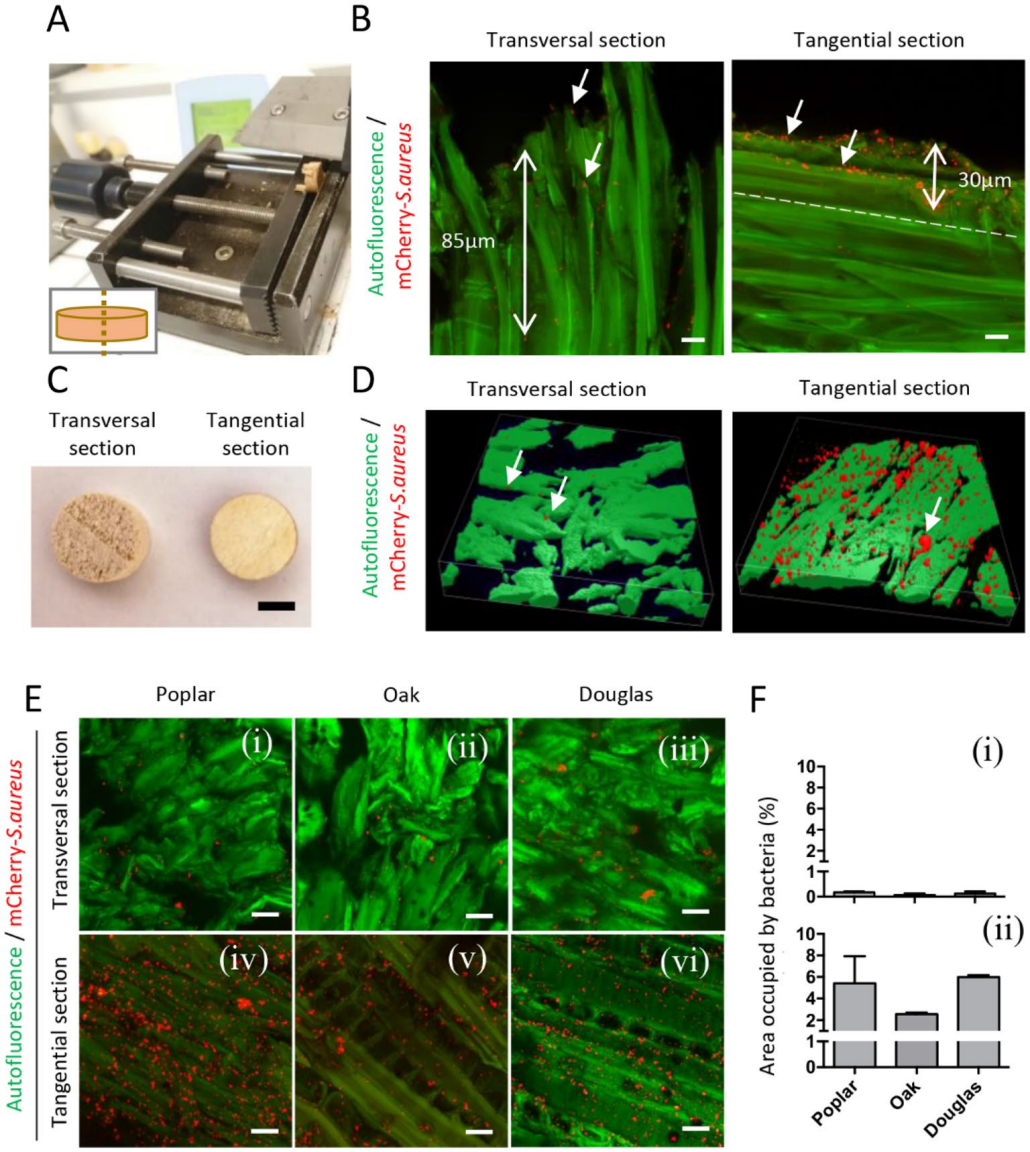
mCherry—*S. aureus* bio-distribution in wood depth and on wood surface. **(A)** Microtome Sect. (50 µm) at the middle of Poplar disc **(B)** mCherry—*S. aureus* (red) bio-distribution in the first 100 µm of poplar disc with cross (left) or longitudinal (right) section. White arrows highlight bacteria spots. Scale bar = 20 µm. **(C)** Non-destructive of transversal (left) and tangential (right) sections poplar disc observations. Scale bar = 500 µm. **(D)** Three-dimensional representation of mCherry—*S. aureus* (red) over poplar (green) with cross (left) and longitudinal (right) section. White arrows indicate mCherry—*S. aureus* spots. 3D dimensions = 140 × 140x30µm. **(E)** Confocal imaging of mCherry—*S. aureus* (red) on transversal (top) and tangential (bottom) sections of poplar, oak and Douglas fir (green; respectively, from left to right) 1-h post-bacteria inoculation. Scale bar = 20 µm. **(F)** Quantification of the surface occupied by mCherry—*S. aureus* (red) related to transversal (top) and tangential (bottom) sections of poplar, oak and Douglas fir area. Error bars represent SEM of the mean.

Two different approaches were investigated to localize mCherry bacteria in wood disc. The first approach was the observation of sectioned inoculated wood by using microtome sectioning. The second approach was the non-destructive direct confocal observation of inoculated discs (Figs. 2, 3).

In the sectioned wooden discs, on the LT section, bacterial penetration is highest at 30 μm from surface to depth and their concentration is higher as compared to RT, which indicates penetration of up to 95 μm (Fig. 3B) with the possible bacteria displacement due to cutting action. Figure 3B is showing almost top 100 μm from surface to depth as comparison of LT and RT microbial distribution. However, the samples were cut through the length and observed total 3–3.5 mm (results not shown). These images are maximum intensity projections obtained from 20 to 25 μm thickness.

The direct observation of inoculated discs revealed rare bacteria on the RT wood surface whereas a high amount of bacteria was observed at the surface of LT wood discs. Images were acquired at least on 30 µm of wood thickness.

Furthermore, a quantitative analysis was done on the collected images by an image analysis software (ImageJ, NIH, US). The results were obtained based on the fluorescence cover of red mCherry spectra as compared to green autofluorescence of wood. It was observed that the bacterial fluorescence covers around 6% of wood surface on LT cutting whereas bacteria represented < 0.1% of the wood surface on RT cutting on all three kinds of woods after inoculation (Fig. 3F).

### Microbial presence on wood on different days

**Figure 4:**
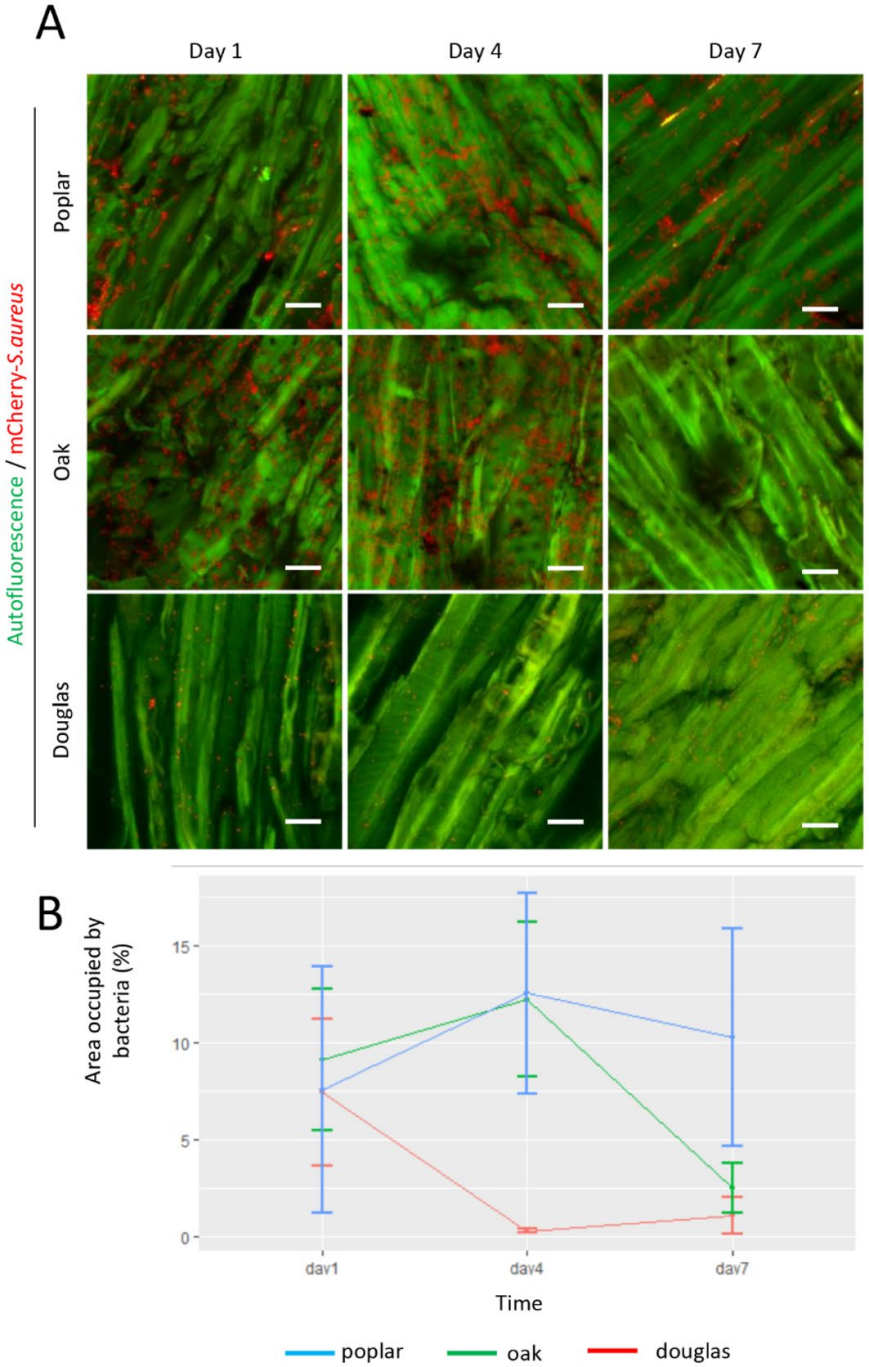
Evolution of mCherry—*S. aureus* bio-distribution on wood surfaces. **(A)** Confocal imaging of mCherry—*S. aureus* (red) on tangential sections of poplar (upper panel), oak (middle panel) and Douglas fir (lower panel) disc at 1-, 4- and 7-days after bacteria inoculation. Woods were observed thanks to their autofluorescence (green). Scale bar = 20 µm. **(B)** Quantification of the surface occupied by mCherry—*S.aureus* spots related to total area of woods. Interactions of time and wood on aera were evaluated by linear mixed effects models considering a random effect (R software, nlme package). Error bars represent SEM of the mean.

As the bacteria tend to remain closer to the surfaces of LT faces, further investigations were done by using this plane. We evaluated the bacterial survival on LT wood surfaces on days one, four and seven for all three wood species. The images collected via CSLM were analysed for bacterial quantity based on the red fluorescent area covered by wood compared to the green area of wood (Fig. 4). The wood area covered by bacteria declined with time depending upon the type of wood material.

In general, the percentage of area covered by red fluorescence for the three wood species on day one was similar (9–13%). Area covered by bacteria decrease more significantly on Douglas than on poplar between 1 and 4 days (p = 0.0017, unilateral test). Furthermore, this area decrease more significantly for Douglas between 1 and 7 days compare to poplar (p = 0.0153). The evolution wood area recovered by bacteria between 1 and 7 days was not significantly different between poplar and oak (p = 0.3452). Area covered by bacteria decreased more significantly on oak compared to poplar between 1 and 7 days (p = 0.0128). Poplar area covered by bacteria stayed high at all time, its change over time was significantly different from other wood species for which area occupied by bacteria trends to decrease with time. Standard deviations were important and therefore limited the significance. Concerning the p-value, the power of the test was between 70 and 72% except for the difference between poplar and Douglas on day 1 and day 4 with a test power equal to 90%.

### Biofilm formation on wood and other surfaces

Understanding biofilm formation in a given situation is necessary concerning surface cleaning studies, especially those with a hygienic perspective.

**Figure 5:**
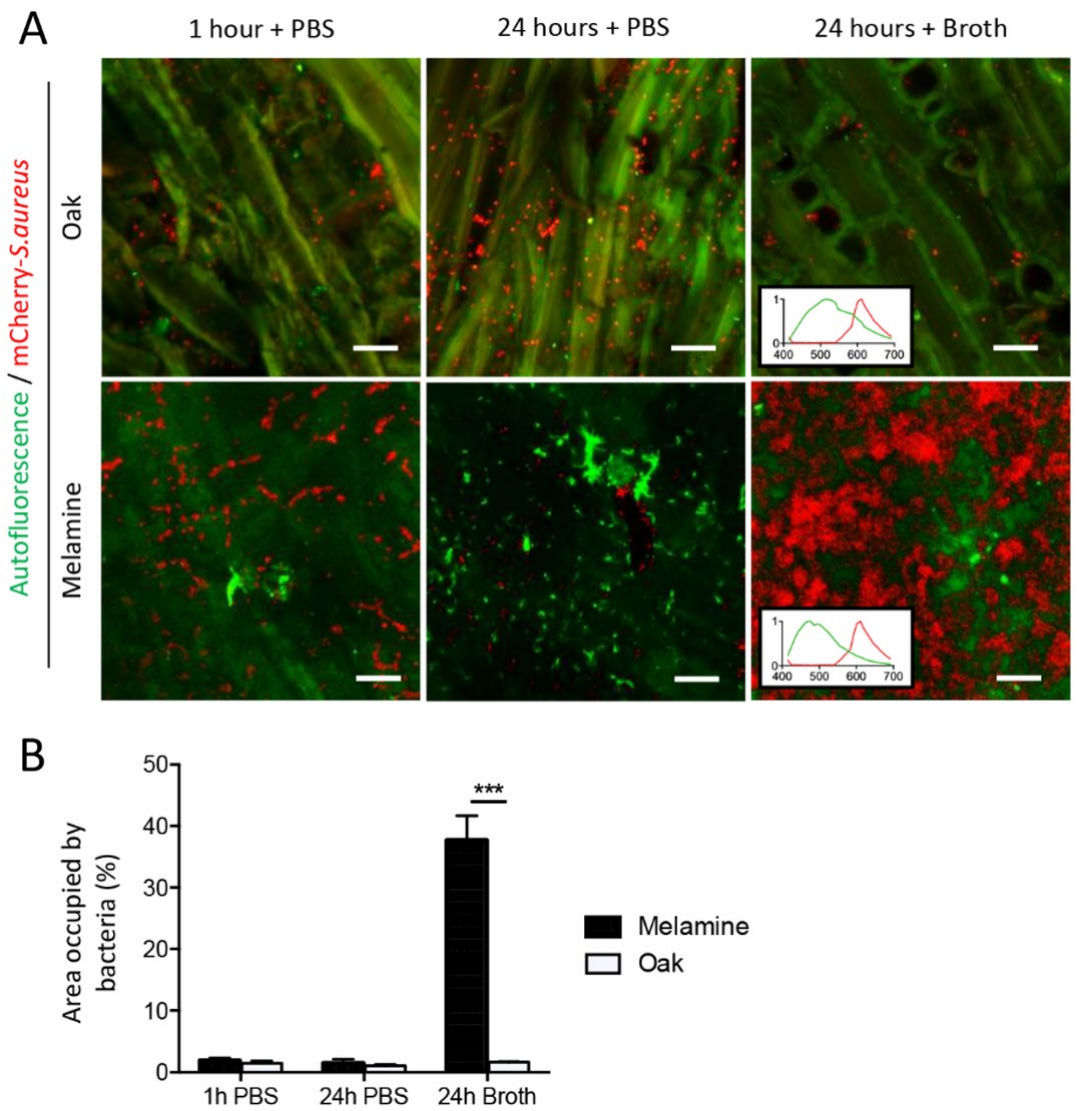
mCherry—*S. aureus* biodistribution on the surface of an oak disc and melamine piece. **(A)** Confocal imaging of mCherry—*S. aureus* (red) on tangential oak disc and melamine piece (green; respectively, upper and lower panel) 1 h, 24 h in presence of PBS and 24 h in presence of broth. Graphics representing autofluorescence of oak and melamine (green) in presence of mCherry (red) are both presented in 24 h both broth pictures. Scale bar = 20 µm. **(B)** Quantification of the surface occupied by mCherry—*S. aureus* spots related to total area of oak and melamine (Two-way ANOVA, Sidak post-hoc test. Error bars represent SEM of the mean. ***p < 0.001).

Trypton soy broth (TSB) was used to improve biofilm formation on oak and melamine surfaces. The CLSM taken images showed that almost equal fluorescence of mCherry is seen on both surfaces after 1 h of incubation (Fig. 5).

The autofluorescence of melamine was less important than autofluorescence observed in wood and we had to use higher power of laser to observe this autofluorescence which was mainly emitted between 400 and 550 nm, highly distinct from red fluorescence of mCherry (Fig. 5). For these reasons, using the melamine autofluorescence was not so essential to differentiate mCherry bacteria from the support. Nevertheless, we decided to use this green autofluorescence to detect the surface of melamine and therefore the biodistribution of red mCherry *S. aureus* on it even this autofluorescence could not be a source of artefact in mCherry fluorescence detection.

Inoculated melamine dishes observed 1 h and 24 h after inoculation did not present the same green autofluorescence pattern on the Fig. 5, probably because the microstructure of this material is not homogeneous and that images were acquired in different area of the discs (we took 10 images by condition). We have also observed green puncta on melamine without bacterial inoculation (results not shown) and on some image obtained from melamine dishes observed 1 h after inoculation (results not shown).

After supplementation with PBS and 24 h of incubation, the quantities of bacteria were similar on both the oak wood and melamine surfaces. However, the interesting finding was that the bacteria formed biofilm and their number increased exponentially on the melamine surface in presence of broth (Fig. 5B). Meanwhile, no such changes were evident on oak wood.

## Discussion

Autofluorescence spectra of poplar, oak and Douglas fir were used for imaging and detection of fluorescent mCherry *S. aureus* on wood surface. High fluorescence intensity of wood was obtained in blue and green from UV excitation probably due to lignin^23,24^*.* Overall, the fluorescence spectra of different species of wood were close. However, some differences in fluorescence spectra were obtained from RT sections in which cytoplasm and cell wall molecules were probably more accessible and source of autofluorescence as flavonoids and pigments^25^. The major blue-green fluorescence emission of wood was the determinant criteria for the choice of fluorescence probe used to stain *S. aureus.* Autofluorescence of melamine was also used to image bacterial bio-distribution on the surface of material with a blue green fluorescence obtained from 405 nm excitation as wood.

The mCherry protein with red fluorescence emission had been previously described in the literature as stable between pH 4 to 10^22^. Therefore, mCherry *S. aureus* was chosen in the present work to investigate red *S. aureus* bio-distribution on green wood and melamine surfaces. A critical point was the high acidic environment of wood^21^, for example, the pH of oak wood is 3.5, which could influence the investigation. However, we have demonstrated that strong acidic pH responsible for green fluorescence switch of mCherry could be avoided by using phosphate buffer for bacterial inoculation and the storage of inoculated wood. In these conditions, the mCherry red fluorescence of *S. aureus* was preserved and could be analysed as representative of bacterial bio-distribution on wood surface that showed a distinct green spectrum from autofluorescence. Previously, published studies have demonstrated the use of fluorescent DNA staining to study bacteria on wood material. Dubreil et al.^17^ used DRAQ5, a DNA probe, to determine the distribution of *Escherichia coli* on poplar wood surface. However, we have tested DRAQ5 labelling on *S. aureus* and this probe was not efficient on these live Gram-positive bacteria^26^ probably due to their bacterial wall highly rich in peptidoglycan which could act as an obstacle to the DRAQ5 staining. Syto9 is another probe described in the literature used to label bacteria fixed on Douglas fir wood surfaces^15^. Nevertheless, we have observed that this green fluorescent nucleic acid stain offered the difficulties to analyze stained bacteria on wood that had high endogenous green autofluorescence (data not shown). Stiefel et al.^27^ identified critical aspects of Syto9 staining *S. aureus* and *Pseudomonas aeruginosa* with bleaching Syto9 over time. Additionnaly, the DNA staining could affect bacterial survival capability^28^. Moreover, direct labelling of bacteria on wood is not highly specific due to the presence of other wood contaminants or wood cells containing DNA. For example, we have observed that DRAQ5 direct staining of wood inoculated with *E. coli* was capable to stain bacteria but also nucleic acid fragments of wood cells^26^. Due to these difficulties, the aforementioned probes are also not optimized to investigate the viability of microbes on wood surface, consequently, the use of mCherry probe helped to overcome these difficulties. On the other hand, van Zyl et al.^29^ have reported an excellent correlation between fluorescence and viable cell numbers, suggesting that photons emitted by the mCherry protein are a good reflection of the metabolic activity of bacteria.

Qualitatively, there was higher red fluorescence on bacteria inoculated LT planes as compared to RT. The first assumption was that the transverse face may have permitted higher diffusion of antimicrobial compounds, as observed in the previous experiments^3,9,10^. However, the more likely reason was the penetration of bacteria into the pores which are more prevalent in the RT plane (Fig. 3). A previous study also reported that a higher number of *E. coli* cells and *B. subtilis* spores were seen on the LT faced wooden cutting boards compared to RT^30^. They observed that bacteria and spores could penetrate further around 3 mm into transverse faces compared to only 1 mm in longitudinal planes of wood. In the present work, the stronger presence of mCherry *S. aureus* at the surface of wood disc with LT orientation is very important data to take into consideration particularly in the hygienic context of wood surface in a hospital. The lower concentration of bacteria on RT surface of wood is presumably due to the transportation of bacteria by the flow of liquid into the porous sample. Therefore, cleaning the wood surface should be more intensive on RT. On the other hand, the persistence of dormant or live bacteria inside porous RT wood could represent a hygienic risk excepted if it is considered that bacteria are stuck inside the complex structures without the possibility of cross-contamination^31–35^. Regarding the existence of porosity on wood material, it is important to mention that during the usage life of wood inside healthcare buildings, the pores are covered by organic debris and bacterial descending deep into wood may diminish^1^. Then, CSLM observations have shown that the orientations of wood LT and RT will be determinant in the *S. aureus* concentration respectively at the wood surface and inside of wood depending on the porosity of sample. In the case of LT wood discs, the bacteria were mainly localized on the first 30 µm into wood structure. These data were obtained from both sectioned wood and 3D representation of un-sectioned LT wood discs (Fig. 3). These findings showed that the CSLM observations of un-sectioned wood discs could be used to study the bacterial contamination on wood surfaces without sample destruction and therefore without artefacts of sectioning operation. This non-destructive method was used not only to observe microbes on wood surface but would be also adapted to melamine, plastic or steel material more or less difficult to be sectioned after bacterial inoculation.

The long-term persistence of *S. aureus* on indoor hospital surfaces has been reported^7,8^. Likewise, the current strain of bacterium showed that it could persist on wood surfaces for more than 1 week. In the present study, we have shown that the number of bacteria decreased throughout the study period on oak and Douglas fir wood surfaces but not on poplar. Our experiments demonstrated for the first time the possibility of direct quantification of bacteria on wood surfaces. The main limitation of current methods used to quantify microbial contamination on surfaces is due to lack of total microbial recovery which happens in the case of swabbing, brushing and elution-based methods^13^. These experiments highlighted the possible effects of wood nature on bacterial contamination and the potential importance of wood structure, as lower bacterial contamination was observed on Douglas fir compared to oak and poplar. Meanwhile, the poplar always showed higher bacterial concentrations as compared to other woods. Both the anatomy and chemical composition of wood could be the influencing factors as they are known to interfere with the microbial survival on the wood material^11^. Moreover, the same microbes are known to have different survival times on different wood species^11^.

*S. aureus* can colonize the inanimate surfaces by forming biofilms in suitable growth conditions in hygienically sensitive places, such as hospitals and the food industry^36^. Biofilms are difficult to clean and can protect the bacterium from disinfectants^37^. Moreover, they do not only increase the chances of microbial survival but also pose a continuous risk of cross-contamination^36^. Therefore, studying the biofilm-forming ability and its characteristics is important to understand the bacterial hygienic risk on surfaces used for food preparation, packaging, cooking, and furniture in hospital or other indoor facilities. In this work, we investigated the biofilm-forming ability of *S. aureus* on oak wood and melamine surfaces. Both these materials are used indoor, especially melamine that is considered as non-porous and easy to clean material. This is the first study that compared the bio-distribution of *S. aureus* on wood and melamine surfaces with the same conditions of bacterial contamination. Although bacterial cells could be seen on the oak sample dosed with TSP or PBS, the biofilm was not formed in both cases. In similar growth conditions, biofilm was observed on melamine with broth. These observations are consistent with previously published data on bacteriostatic or bactericidal properties of wood^5,9,11,13^ and strengthened the interest in wood introduction compared to melamine material.

The formation of biofilm on melamine and not on wood opens new queries regarding the hygienic nature of nonporous materials which are generally considered as more hygienic as compared to porous materials like wood. In these situations, if wood is considered more hygienic compared to melamine, numerous new questions are raised about using wood in hygienically sensitive places.

The CSLM is generally used for observing biofilms in solution and on smooth solid surfaces. On wood, CSLM is generally used to study the topography and surface roughness^38^. However, it can give promising results when used to observe bacterial colonization on wood surfaces. Dubreil et al.^17^ used DRAQ5 to observe *E. coli* on wood surface by using CLSM, Lortal et al.^15^ used confocal microscopy and syto9 to observe yeast and bacterial biofilms on Douglas fir wood used in the cheese industry. Our innovative approach used genetically modified *S. aureus* with 100% of mCherry expression and fluorescence spectra highly distinct to wood and melamine autofluorescence, thus allowing direct quantification of bacteria on raw materials. However, we have to acknowledge that we cannot rule out that there were dead bacteria among mCherry bacteria detected in and on wood, this will be the subject of future work.

This type of demonstration can be a very promising tool to determine the real distribution of large genetically modified bacteria species on raw materials, especially for hygiene education.

## Conclusions

The non-destructive (no material sectioning) and label-free imaging approach (no wood staining) by using UV excitation of natural and synthetic material is an original method to study and compare mCherry microbe bio-distribution on different material to give essential information on hygienic properties of raw and in use material. The mCherry *S. aureus* was concentrated at the surface of wood disc with LT direction, most commonly used for making laminate flooring, furniture, and food packaging surfaces whereas bacteria diffused inside the porous structure of wood disc with RT direction mainly used to prepare the cutting boards and flooring tiles.

The qualitative and quantitative results showed that the Douglas fir was the least colonized, while the poplar was the most colonized wood. In addition, bacterial numbers decreased gradually on wood from day four to seven but were still present one week after inoculation. Finally, the experiment showed that the plasmidic mCherry *S. aureus* strain formed a dense biofilm on a melamine surface 24 h post bacterial inoculation in the presence of broth whereas biofilm was not formed on the oak wood in similar experimental conditions. These interesting results are counterintuitive to the general perception that wood supports the biofilm formation and therefore not used in hygienically sensitive places; meanwhile, melamine is abundantly used as a contact surface in hygienically sensitive places.

## Materials and methods

### Wood and melamine

Sessile oak (*Quercus petraea*), Douglas fir (*Pseudotsuga menziesii*) and poplar (*Populus euramericana alba* L.) wood species were selected for this study. Oak is used for indoor construction and furniture making due to its durability and known antimicrobial properties^3^. Douglas fir wood is also used for indoor constructions but mostly outdoor applications and food preparation surfaces^10,39^. Poplar wood is used for food packaging and processing material^40^. The samples were obtained directly from sawmill and originated from vicinities around Nantes, France. The logs were naturally dried for three months and the samples were cut from heartwood in the form of round wood discs (diameter 9.95 ± 0.1 mm, thickness 3.5 ± 0.4 mm) of transversal (RT) and tangential faces (LT)^9,41^. Melamine impregnated paper was obtained from the workshop of Ecole Supèrieure du Bois, Nantes. Melamine is a synthetic material that is extensively used as a contact surface in healthcare buildings^42^.

### mCherry bacteria and culture conditions

Genetically modified fluorescent strains of *S. aureus* with mCherry expressing gene inserted in their plasmid (Plasmidic mCherry label, LUG2929: SH100/pLUG1315: pSK:: PsarA-mCherry) was obtained from CNR *Staphylococcus aureus* (CNR : Centre National de Référence), Lyon, France, courtesy of Dr. Jessica Baude. Previously, it has been reported that mCherry gene does not interfere with microbial growth^29^. This probe was selected instead of conventional green fluorescent protein (GFP) because of its higher stability in the acidic environment^22^ since most wood species have an acidic pH^21^. Another reason was that the fluorescence emission of wood was mainly in the green channel which would be very difficult to differentiate from that of GFP. The plasmidic strain was pre-cultured on plate count agar (PCA) and then in Tryptic soy broth (TSB) containing chloramphenicol (10 μg/ml), with incubation at 37 °C for 24 h. This strain was coagulase-positive and had a peak growth curve time at 15 to 16 h.

### Inoculation and incubation

The bacteria were harvested from the fresh culture as described by Khelissa et al.^43^. Briefly, the TSB broth culture was centrifuged at 5000 g for 5 min at 20 °C. Cells were washed twice and suspended in 20 ml of phosphate buffer saline (PBS—pH 7.4). The suspension was vortexed for one minute and then sonicated at 65 Hz for 30 s to attain a homogenous dispersion. The turbidity of suspension was adjusted to 1.5–2 McFarland and the colony-forming units (CFU) were determined by plate count method^5^. A 50 μl volume of the PBS based bacterial suspension was inoculated on the centre of wooden discs and incubated at room temperature (20–22 °C). The inoculated dishes were covered by aluminum foil to protect fluorescent probe from light. Inoculations were realized on wood discs of three wood species with two cutting directions, LT and RT sections at four incubation times including 1 h, 24 h, 4 and 7 days. After the specific incubation time, the wooden discs were immersed in Glutaraldehyde (0.5% in PBS) for ten minutes at room temperature (20 °C) and washed thrice with PBS. Finally, the discs were submerged in the PBS in a 12 well plate and stored at 4 °C until further observation. CSLM imaging was performed on the surface and at the middle section of wood discs.

For the biofilm studies, the inoculated material pieces were rinsed with distilled water one hour after inoculation to remove the unattached bacteria. Furthermore, the surface of each material was covered with 2 ml of TSB (Liofilchem, Italy) or with 2 ml of normal saline (NS—Physiodose + , Gilbert). After 24 h of incubation, the liquid medium was gently removed, and the samples were rinsed with PBS and fixed according to the above-mentioned methodology. All this process took place in the laminar flow cabinet at room temperature (~ 22 °C).

### Confocal spectral laser microscopy (CSLM): image acquisition and analysis

The autofluorescence spectra of LT and RT cuttings of three wood (oak, Douglas fir and poplar) were obtained by using CSLM (Zeiss LSM 780, Germany) and recorded.

A stack of at least 30 images (one image every 1 µm) was done for each acquisition. Three to ten acquisitions were done per wood specimen placed on a drop of PBS on a glass slide. The individual fluorescence signals of different products (wood, bacteria and melamine) were captured by using 32 GaAsps detectors which were capable of detecting fluorescence between 405 and 665 nm in spectral mode. Two to three mean spectra representative of wood autofluorescence were acquired for each wood species from tangential and transversal discs, these spectra were used for the online finger printing operation.

The dual sequential laser excitation approach with 405 nm and 561 nm beam laser was adapted to observe both fluorescent signals generating by mCherry bacteria (561 nm excitation) and the autofluorescence (405 nm excitation) of material which were unmixed by using linear separation technique^44^. Separation of spectra was done by using a highly sensitive online fingerprinting module (LMS780, Zeiss).

We highlighted the different spectral signature for oak for which presence of “high amount of extractible” have been already described. And we thought that these extractives could be assigned to blue fluorescence of wood. So for online finger printings we took care to use spectra representative of the each wood. We used at least two mean spectra of autofluorescence by wood species and a red spectra of m Cherry *S. aureus* obtained from mCherry bacterial solution. Online fingerprinting was realized by using spectra obtained from each wood autofluorescence which were associated to green color and the spectra of mCherry *S. aureus* which was associated to red color. It means that each pixel which was composed by spectra corresponding to autofluorescence was in green color and each pixel composed by spectra of mCherry was in red color. The fluorescence spectra between autofluorescence and mCherry was highly distinct.

A maximal z projection was realised from each stack of 30 images. No red fluorescence was detected after fingerprinting operation on control wood which were wood dishes without any bacterial inoculation.

We used ImageJ software to do image analysis. To count mCherry *S. aureus*, (1) background due to autofluorescence was removed (2) a common threshold value was fixed to consider the red positive signal, (3) a segmentation and a binarisation were performed to obtained the value of area covered by red fluorescence. Red area was reported to the green area corresponding to material surface. These operations were automatised by writing a macro. The thresholding was validated on images obtained from control wood sample (without bacterial inoculation). Counting bacteria cells in maximum intensity projections of the original data may introduce artefacts or uncertainty in the measurement. At least three distinct experiments were done with five to ten images in each condition.

### Statistical analysis

Interactions of time and wood on aera were evaluated by linear mixed effects models considering a random effect (R software, nlme package). Two-way ANOVA, Sidak post-hoc test was used to compare the percentage of area recovered by bacteria on wood and melamin from 1 to 24 h in presence of PBS or with broth.
